# 

**Published:** 1896-09

**Authors:** 


### CONTENTS.

COMMUNICATIONS.

Lining Root-Canals........................................................ 417
Materia Medica and Therapeutics........................................ 422
The American Medical Association....................................... 427

PROCEEDINGS.

National Association of Dental Faculties.............................. 449

SELECTIONS.

Kerosene in Surgery........................................................ 457
Digestible Food............................................................. 458
Asbestos as a Surgical Dressing......................................... 459
Facial Neuralgia............................................................ 459
Local Anæsthetic........................................................... 459
Chloral........................................................................ 459
Capacity of Women for Bearing Pain.................................... 460
Quantity of Food........................................................... 461
The Nerves of Taste........................................................ 463

EDITORIAL.

American Dental Association.............................................. 464
National Association of Dental Faculties.............................. 465
Ohio State Dental Society.................................................. 467

